# Genomic Regions Identified by Overlapping Clusters of Nominally-Positive SNPs from Genome-Wide Studies of Alcohol and Illegal Substance Dependence

**DOI:** 10.1371/journal.pone.0019210

**Published:** 2011-07-27

**Authors:** Catherine Johnson, Tomas Drgon, Donna Walther, George R. Uhl

**Affiliations:** Molecular Neurobiology Branch, National Institutes of Health, Intramural Research Program, National Institutes on Drug Abuse, Baltimore, Maryland, United States of America; Aarhus University, Denmark

## Abstract

Declaring “replication” from results of genome wide association (GWA) studies is straightforward when major gene effects provide genome-wide significance for association of the same allele of the same SNP in each of multiple independent samples. However, such unambiguous replication is unlikely when phenotypes display polygenic genetic architecture, allelic heterogeneity, locus heterogeneity and when different samples display linkage disequilibria with different fine structures. We seek chromosomal regions that are tagged by clustered SNPs that display nominally-significant association in each of several independent samples. This approach provides one “nontemplate” approach to identifying overall replication of groups of GWA results in the face of difficult genetic architectures. We apply this strategy to 1 M SNP GWA results for dependence on: a) alcohol (including many individuals with dependence on other addictive substances) and b) at least one illegal substance (including many individuals dependent on alcohol). This approach provides high confidence in rejecting the null hypothesis that chance alone accounts for the extent to which clustered, nominally-significant SNPs from samples of the same racial/ethnic background identify the same sets of chromosomal regions. It identifies several genes that are also reported in other independent alcohol-dependence GWA datasets. There is more modest confidence in: a) identification of individual chromosomal regions and genes that are not also identified by data from other independent samples, b) the more modest overlap between results from samples of different racial/ethnic backgrounds and c) the extent to which any gene not identified herein is excluded, since the power of each of these individual samples is modest. Nevertheless, the strong overlap identified among the samples with similar racial/ethnic backgrounds supports contributions to individual differences in vulnerability to addictions that come from newer allelic variants that are common in subsets of current humans.

## Introduction

Genome wide association (GWA) is a method of choice for identifying genes whose variants influence vulnerability to complex disorders. Declaring “replication” of individual results of genome wide association studies is straightforward when major gene effects provide associations between marker and phenotype that display the same phase and “genome wide” levels of significance (p *ca* 10^−8^) in each of several independent samples. However, such “template” replication for individual markers is unlikely to be achieved in many otherwise-reasonable samples for many phenotypes. Phenotypes and samples that display polygenic genetic architecture, allelic heterogeneity, locus heterogeneity and sample-to-sample differences in fine structures of linkage disequilibrium can provide especial difficulties for this “template” approach. These difficulties can be exacerbated when data comes from different genotyping platforms that do not assess allele frequencies for identical sets of SNPs. Much current genome wide association and linkage data suggests that we may have identified many or even most of the loci at which we might expect “template” analyses to identify reproducible genome wide significance in reasonably sized samples *(see references below)*. Much of the risk attributable to genetic influences on common phenotypes appears likely to arise from polygenic influences whose properties are likely to provide many false negative results in searches for replicated “genome wide” significance in multiple independent samples that use “template” criteria for replication.

Vulnerability to heavy use and development of dependence on alcohol and/or an illegal abused substance (“addiction vulnerability”) appears to be such a trait. The substantial genetic influences on addiction vulnerability are documented by data from family, adoption and twin studies [1], [2], [3], [4]. Twin studies also document shared heritable influences on vulnerability to dependence on addictive substances from different pharmacological classes, including alcohol and illegal drugs from several pharmacological classes [2], [3], [5]. Combined data from linkage and initial GWA studies [6], [7], [8], [9], [10], [11], [12], [13], [14], [15], [16], [17], [18], [19] suggest that much of the genetic influence on vulnerability to substance dependence is likely to be polygenic.

We have developed a “nontemplate” strategy that identifies overall replication of *sets* of genome wide association (GWA) results in the face of difficulties with genetic architectures, samples and genotyping methods [9], [14], [20], [21]. Such an approach can complement meta-analyses that seek to combine data from single markers whose significance in single samples does not achieve genome wide significance.

We now report application of this nontemplate strategy to identify overall replication of groups of results from GWA studies of samples of individuals with dependence on alcohol and illegal substances *vs* matched controls [21], (http://www.ncbi.nlm. nih.gov/gap). We separately compare data from independent samples of individuals with European-American genetic backgrounds and samples of individuals with African-American genetic backgrounds. These data come from individual genotyping and multiple-pool genotyping approaches that use 1 M SNP Illumina and Affymetrix platforms, respectively. The results focus attention on chromosomal regions that are identified by clusters of SNPs for which case *vs* control differences achieve nominal statistical significance in multiple samples from the same racial/ethnic group. We describe the high confidence with which this approach rejects the null hypothesis that clusters of nominally-significant SNPs from different samples of individuals from the same racial/ethnic group identify the same chromosomal regions with frequencies expected by chance. We note the more modest levels of confidence that this approach provides for identification of individual SNPs, individual chromosomal regions, individual genes and for the overlap between data from samples of the two racial/ethnic groups studied, except in genes in which we and other investigators have identified associations in independent samples. We discuss this work in light of its technical and analytic limitations and in its similarities with and differences from “template” GWA analyses and meta-analyses that seek reproducible associations of striking levels of significance at single SNP markers. The current “nontemplate” replication of sets of results may be useful in other settings in which the underlying properties of the disorder and of the samples create difficulties for searches for individual SNPs with replicated genome wide significance.

## Materials and Methods

### Subjects, genotyping and assignment of nominal significance of dependent vs control allele frequencies in each sample

#### 1) dbGAP samples from the FSCD, COGA and COGEND studies

Genotypes from unrelated subjects who provided written consents and met DSM criteria for alcohol dependence and consenting control subjects with no evidence for dependence on any drug were assembled from three sets of subjects and deposited in dbGAP (http://www.ncbi.nlm.nih.gov/projects/gap/cgi-bin/study.cgi?study_id=phs000092.v1.p1). Family study of cocaine dependence (FSCD) subjects were recruited from treatment centers close to St. Louis. Mo; 55% of contacted subjects participated [22]. Community-based comparison subjects were recruited through driver's license records from the Missouri Family Registry and were matched to alcohol dependent subjects based on date of birth, ethnicity, gender, and zip code. Eighty percent of screened and eligible comparison subjects participated. Other participants came from individuals who participated in the Collaborative Study on the Genetics of Alcoholism (COGA) [23] and the Collaborative Study on the Genetics of Nicotine Dependence [10]. Dependent individuals displayed DSM (Diagnostic and Statistical Manual IV) dependence on alcohol. Controls, defined in dbGap variable phv00022939.v1.p1.c2 “final_type”, displayed no DSM dependence on alcohol, cocaine, marijuana, opioids or other drugs but may have evinced DSM nicotine dependence, FTND scores >4 and/or regular smoking as defined by smoking >100 cigarettes in their lives. We identified 1171 dependent and 1395 control unrelated European-American subjects and 652 dependent and 499 control unrelated African-American subjects for this analysis. Subjects were 45% male; 48% of the alcohol-dependent subjects were also dependent on cocaine.

Genotyping for these samples was performed using Illumina 1 M SNP arrays at the Center for Inherited Disease Research (CIDR), with quality controls and principal components analysis (PCA) controls for racial/ethnic background available at the dbGAP website. Genotypes from dependent and control individuals were selected from dbGAP files, excluding SNPs with minor allele frequencies less than 0.01–0.02 (for European and African American samples, respectively) and those with missing call rates >5%. p values for each SNP were based on χ^2^ tests.

#### 2) NIDA/MNB samples

European-American and African-American research volunteers, largely non treatment seeking, came to the NIDA research facility in Baltimore, Maryland between 1990 and 2007 in response to advertisements and referrals from other research volunteers. Subjects provided written informed consents, self-reported ethnicity data, drug use histories *via* the Drug Use Survey and DSMIII-R or IV diagnoses (Diagnostic and Statistical Manual) and were reimbursed for their time as previously described [6], [17], [21], [24]. Genotypes were assessed in DNA pools using Affymetrix 6.0 arrays and methods that we have extensively validated, as previously described [6], [7], [8], [9], [21]. Pooling 1) provided us with the maximal ability to protect the genetic confidentiality of subjects who volunteered for study of genetics of illegal behaviors, 2) allowed us to utilize DNAs from individuals who consented to participation in this study during time periods when consents did not explicitly describe studies using high densities of DNA markers, 3) allowed us to use methods that we have developed and validated in this and in previous work and 4) reduced costs. Many of these subjects would thus not have been available for studies that assessed substantial numbers of polymorphisms using individual genotyping. Nominal p values for each SNP were determined based on t tests that compared data from multiple abuser *vs* control pools that contained DNAs from 680 European-American and 940 African-American individuals who had mean ages of 32.8 and 34.0 and were 69.5 and 58.8% male, respectively, as described [21]. In addition, to provide additional validation for the pooling results for the SNPs that formed the basis of the clusters evaluated herein, we also performed individual genotyping using Affymetrix 6.0 arrays for the 155 African American research volunteers who constituted virtually all of the members of 8 DNA pools and who had consented to unlimited individual genotyping. These individual genotyping results all passed Affymetrix quality control standards and resulted in ≥98% call rates.

#### 3) Identification of chromosomal regions containing clusters of SNPs with nominally-significant case vs control differences in single or multiple samples

We performed analyses based on previously-defined criteria using datasets of approximately 1 million SNPs [21]. We identified chromosomal regions of interest in individual samples by seeking regions in which at least 4 clustered SNPs displayed case *vs* control differences with nominal, p<0.05 levels of statistical significance. We defined clustering based on separation of each clustered SNP from the nearest nominally-significant SNP by ≤10 kb. We identified similarities between the results obtained from multiple samples by identifying the chromosomal regions that were tagged by such clustered, nominally positive SNPs in each of the samples of individuals from the same racial/ethnic groups. We identified genes for which these chromosomal intervals lay within the exons of the gene and/or in 10 kb of 5′ or 3′ flanking sequence.

#### 4) Monte Carlo methods for assignment of levels of significance to: a) the extent of clustering in each sample and b) the degree to which clustered nominally-positive SNPs from multiple independent samples identify the same chromosomal regions

Monte Carlo methods were used to assign empirical statistical probabilities to two null hypotheses, starting with the sets of all SNPs and the nominally positive SNPs that displayed p<0.05 case *vs* control values.

We first tested the null hypothesis that chromosomal clustering of these nominally positive SNPs occurred at the level expected by chance in these datasets. For each Monte Carlo trial that tested this null hypothesis, we randomly selected a number of “pseudo positive” SNPs from each dataset that matched the number that achieved nominal significance in the *bona fide* dataset. Thus, we constructed a list of autosomal SNPs assayed in each sample and assigned a number to each SNP that corresponded to its position on the list. To select the pseudopositive SNPs for each trial of the European-American datasets, we selected 75,413 random numbers for the NIDA *(see below)* and 49,843 random numbers for the dbGAP datasets. For the African American datasets, we used 83,330 and 45,325 random numbers, respectively. For each trial, the SNPs identified by the positions on the list that corresponded to these randomly-assigned numbers were then queried for the extent to which their results equaled or exceeded the results obtained for the actual dataset. In 10,000 such trials for each sample, we compared results concerning the extent of chromosomal clustering from these sets of pseudopositive SNPs to those for the true positive SNPs. These empirical Monte Carlo p values thus addressed the null hypothesis that the true positive SNPs from each single sample were randomly arrayed on the chromosomes. Of course, the clustering of SNPs that provided nominally-significant case *vs* control differences in each individual sample did not allow us to discern whether the haplotypes identified in such a manner were related to a) phenotypic differences or to b) stochastic differences in haplotype frequencies between case and control samples.

Monte Carlo methods were also used to assign empirical statistical probabilities to a second null hypotheses: that the same chromosomal regions were identified by the clustered, nominally positive SNPs in independent samples with the frequencies expected by chance. In 10,000 trials from pairs of independent samples, we compared the extent of overlap between the chromosomal regions identified by the clustered, nominally-positive SNPs in each sample. The Monte Carlo p values that derive from these trials thus addressed the second null hypothesis that the chromosomal regions identified by clusters of nominally positive SNPs in each of multiple samples were identified only on stochastic bases that were unrelated to phenotype.

Secondary analysis of dbGAP data used permutation approaches as implemented in PLINK (v1.06) (http://pngu.mgh.harvard.edu/purcell/plink/) [25]. We randomized assignment of the phenotypes to data derived from the current SNPs and analyzed the data from 3,000 permutation trials that addressed each of several null hypotheses *(see below)*.

To assess the power of our current approach we used current sample sizes and standard deviations, power calculator PS v2.1.31 [26], [27] and α = 0.05.

## Results

As noted elsewhere [21], variation among the allele frequency estimates between pools from individuals of the same phenotype for each racial/ethnic group from the NIDA/MNB samples was +/− 0.02 (standard error of the mean SEM).

### European-American samples

For the dbGAP data from European-Americans, χ^2^ tests displayed p<0.05 for 49,843 autosomal Illumina SNPs. For the NIDA/MNB European-American samples, 75,413 of the autosomal Affymetrix 6.0 SNPs displayed t values with p<0.05 in comparisons between data from substance dependent *vs* control samples [21].

### Searches for genome wide significance in each European-American sample

We identified case *vs* control p values for t test results from NIDA/MNB samples and for χ^2^ results from dbGAP samples from unrelated individuals. Permutation testing for the dbGAP European-American samples revealed p<0.0003 (3,000 trials) for the number of SNPs with nominal case *vs* control p values<0.05. However, virtually none of these p values reached the 10^−8^ level deemed necessary for genome wide significance.

### Searches for clustering of SNPs with nominally-significant case vs control differences in each European-American sample

We identified 3125 clusters of SNPs that displayed nominally significant, p<0.05 case *vs* control differences for χ^2^ results from dbGAP samples and 2931 clusters with nominally significant t test results from NIDA/MNB samples.

### Searches for chromosomal regions identified by clustered SNPs with nominally-significant case vs control differences in both European-American samples

Two hundred four chromosomal regions contained clusters of nominally-significant SNPs from both of these two European-American samples.

None of 10,000 Monte Carlo simulation trials that each began with random sets of SNPs selected from each of the datasets identified as many overlapping regions as found in the true dataset. The overall Monte Carlo p<0.0001 for the overlap noted in the true data thus provides very high levels of confidence that these independently-derived sets of results do not identify the same set of chromosomal regions by chance alone. Thus, the null hypothesis that the chromosomal regions identified by both samples are identified based only on stochastic grounds is falsified by these Monte Carlo data.

In addition, none of 3,000 permutation trials provides data that identifies as many chromosomal regions from permutated data as those identified by the real datasets. Thus, the null hypothesis that the chromosomal regions identified by both samples are identified based only on stochastic grounds also nullified by permutation testing data. The genes that: a) lie in chromosomal regions identified by data from both European-American samples and b) display the most nominally-significant SNPs are listed in Table 1; the complete list of chromosomal regions identified in this way is listed in Table S1. The fraction of the genome occupied by these results is 210% of the size expected by chance, based on the fractions of the genome occupied by clustered nominally positive results from each of these two European-American samples *(data not shown)*.

**Table 1:** Chromosomal regions and genes identified by clusters of SNPs that provide nominally-significant differences between individuals dependent on alcohol (dbGAP alcohol dependent *v* ctl) or at least one illegal substance (NIDA/MNB drug dependent v ctl) in subjects of European-American heritage.

	dbGAP alcohol dependent v ctl					NIDA/MNB drug dependent v ctl					
*ch*	*# SNPs*	*bp:start*	*bp:end*	p_min_ SNP	p_min_	*# SNPs*	*bp:begin*	*bp:end*	p_min_ SNP	p_min_	gene(s)
1	4	20008199	20013168	rs3820317	9.84E-03	4	20012477	20016730	rs11810916	1.09E-02	RNF186
1	7	55288592	55299911	rs483462	7.99E-04	5	55297559	55314269	rs12118986	1.60E-03	PCSK9, USP24
1	8	55310973	55329655	rs683880	5.67E-04	5	55297559	55314269	rs12118986	1.60E-03	PCSK9, USP24
1	6	65809029	65825669	rs11208674	1.36E-02	4	65806284	65815607	rs1749499	7.20E-03	LEPR
1	4	156627725	156642253	rs12756570	3.68E-03	4	156619836	156635649	rs4661129	3.97E-03	OR10T2
1	9	166920481	166942595	rs524705	4.40E-03	9	166920955	166961651	rs577317	2.28E-04	DPT
1	6	166957917	166974598	rs1052591	5.06E-03	9	166920955	166961651	rs577317	2.28E-04	DPT
1	12	170613171	170634300	rs2227198	2.21E-04	7	170608822	170631953	rs12145969	1.65E-03	DNM3
1	4	177978272	177994423	rs1052447	2.61E-03	5	177993055	178013737	rs1754352	6.97E-03	C1orf76
1	8	199376526	199391033	rs6694122	2.04E-04	4	199383345	199385625	rs7541884	5.23E-03	TMEM9
1	9	229429623	229483170	rs16854012	6.47E-03	4	229444384	229460142	rs4567343	2.74E-03	C1orf131, GNPAT
1	12	243603976	243639175	rs1173837	5.29E-03	7	243627708	243641337	rs962786	7.14E-03	KIF26B
1	15	244056488	244072421	rs9728248	1.84E-04	4	244061674	244078022	rs780240	4.53E-03	SMYD3
2	7	19927818	19951465	rs11096626	6.28E-03	6	19936320	19953950	rs6709385	3.04E-03	TTC32
2	11	38250946	38280657	rs183487	6.45E-04	4	38269132	38283958	rs17014705	2.46E-03	C2orf58
2	4	166876339	166885605	rs4438497	6.47E-03	5	166871909	166879436	rs12712157	1.33E-03	SCN9A
2	16	233405444	233445376	rs2675966	2.84E-03	5	233429928	233444593	rs955944	2.84E-04	NGEF, TNRC15, UNQ830
3	12	7136276	7170141	rs1353828	5.56E-03	5	7166923	7178151	rs16865440	6.74E-03	GRM7
3	27	10923486	11013807	rs4684746	1.94E-04	7	10943172	10961153	rs17583433	6.13E-03	SLC6A11
3	4	15270368	15283244	rs1318937	1.44E-04	4	15274441	15283244	rs12473173	1.31E-02	CAPN7, SH3BP5
3	12	29382520	29425986	rs13084147	2.17E-03	9	29407297	29432158	rs2700165	3.91E-03	RBMS3
3	9	37924180	37957695	rs9822761	3.39E-03	7	37935049	37958835	rs6710782	1.79E-03	CTDSPL
3	5	62634698	62643369	rs17356252	1.31E-03	4	62625164	62637893	rs11563201	1.59E-02	CADPS
3	6	144082777	144094347	rs6778966	3.11E-03	4	144085356	144087277	rs1513215	1.22E-02	PCOLCE2
4	6	5478592	5489229	rs4017782	1.70E-02	4	5485061	5489334	rs6809002	2.83E-03	STK32B
4	7	6092645	6116331	rs6850751	3.02E-03	10	6083725	6111793	rs4574309	2.61E-03	JAKMIP1
4	4	20933289	20944461	rs17520130	1.52E-02	4	20943383	20960603	rs13316480	9.42E-03	KCNIP4
4	7	22037427	22051143	rs1463000	2.15E-03	5	22041791	22059150	rs2350488	2.20E-04	GPR125
4	4	54659085	54667957	rs2278141	4.16E-03	4	54661971	54671211	rs7650251	3.75E-03	GSX2
4	5	90141166	90161886	rs1795722	9.59E-04	4	90140097	90149157	rs2903643	2.72E-03	FAM13A1
4	14	95774856	95821573	rs11724023	3.68E-04	4	95807472	95811400	rs10027043	1.86E-03	PDLIM5
4	7	148002578	148028761	rs1396716	1.04E-03	4	147999449	148013733	rs17587144	6.65E-05	TTC29
4	4	178492741	178506247	rs7689099	2.76E-02	5	178492904	178512644	rs11731709	3.30E-04	NEIL3
4	11	185544039	185583197	rs724528	8.75E-04	11	185570737	185604825	rs17148190	2.78E-03	IRF2
4	5	187423999	187443174	rs4241824	2.14E-03	5	187416129	187441931	rs10518112	5.31E-04	F11, KLKB1
5	7	16733772	16752512	rs2288433	1.71E-02	9	16737109	16755375	rs10019942	1.17E-02	MYO10
5	7	60877967	60899218	rs1550816	2.10E-02	4	60883961	60898674	rs7676941	2.72E-03	ZSWIM6
5	5	78287103	78299426	rs921945	2.12E-02	10	78250443	78293007	rs10866307	1.77E-02	ARSB
5	18	96108157	96159991	rs30333	3.95E-03	4	96108387	96124514	rs2935598	4.75E-03	CAST, ERAP1
5	18	96108157	96159991	rs30333	3.95E-03	5	96138710	96151541	rs12187040	3.59E-03	CAST, ERAP1
5	4	107328554	107341302	rs10900900	2.59E-02	6	107334222	107357187	rs7710617	4.20E-03	FBXL17
5	9	156312645	156338524	rs6883317	8.94E-03	5	156313948	156338362	rs16894458	0.03297	TIMD4
5	8	169753936	169783643	rs13175143	7.58E-04	4	169755299	169768102	rs298387	1.00E-02	KCNIP1, KCNMB1
6	8	125611562	125629660	rs3799732	3.45E-03	7	125615011	125629660	rs1010284	1.03E-03	HDDC2, TPD52L1
6	5	128874118	128891370	rs17364118	1.09E-02	4	128869961	128879810	rs1997781	1.02E-02	PTPRK
6	10	152750003	152780038	rs214989	2.46E-04	5	152756299	152775286	rs4143334	7.29E-03	SYNE1
6	5	167036150	167052518	rs4710081	3.99E-03	6	167007415	167038192	rs16896407	2.82E-03	RPS6KA2
7	7	11485443	11520621	rs6972615	2.45E-03	6	11479011	11498204	rs9449067	2.42E-03	THSD7A
7	8	21647276	21678449	rs6461593	3.91E-03	4	21652526	21661805	rs1020320	7.00E-04	DNAH11
7	5	29478611	29483175	rs1362364	2.94E-02	5	29472600	29486168	rs589469	1.10E-02	CHN2
7	8	50468934	50492914	rs963739	6.76E-04	11	50491702	50517353	rs9398913	1.14E-02	DDC, FIGNL1
7	4	50623828	50640401	rs12540874	9.46E-04	9	50624515	50661951	rs17060099	5.62E-03	GRB10
7	5	50653096	50663588	rs980716	3.29E-03	9	50624515	50661951	rs17060099	5.62E-03	GRB10
7	14	149948324	150001479	rs6946579	5.64E-03	4	149950223	149957752	rs11972731	2.46E-03	GIMAP6
7	4	154283212	154287670	rs878742	1.06E-02	4	154278320	154298576	rs10237037	5.03E-03	DPP6
8	6	3030719	3047485	rs1077153	6.75E-04	4	3035516	3042040	rs6942789	2.68E-02	CSMD1
8	10	3492983	3504244	rs2469390	1.37E-03	5	3491640	3497769	rs12699472	4.43E-03	CSMD1
8	4	3925003	3934454	rs1971078	2.50E-02	9	3907290	3934466	rs7789550	7.17E-03	CSMD1
8	4	4173158	4179668	rs1847570	1.68E-02	7	4166391	4177720	rs7804595	1.56E-05	CSMD1
8	5	10426159	10433464	rs7008087	1.39E-02	4	10415815	10426892	rs4385377	7.64E-03	UNQ9391
8	10	17198427	17226143	rs7003503	3.03E-03	10	17177416	17230166	rs12538892	2.44E-03	MTMR7, VPS37A
9	4	4193214	4216155	rs10974390	1.37E-02	4	4193214	4196671	rs341676	8.82E-03	GLIS3
9	6	100869363	100891728	rs1537504	5.46E-03	6	100853373	100876607	rs4237043	6.49E-04	COL15A1
9	5	111559709	111564648	rs2025878	3.23E-03	6	111545414	111562096	rs16938588	9.40E-03	PALM2
9	10	124177516	124204757	rs10513402	9.32E-03	5	124194135	124204757	rs2319361	1.32E-02	PTGS1
9	4	128199379	128209802	rs4836537	2.19E-02	4	128187181	128205955	rs6982224	4.57E-03	FAM125B
9	4	129336305	129348849	rs1891730	1.49E-02	4	129346310	129350356	rs16895390	7.01E-03	FAM129B
10	8	14397705	14415265	rs7082219	1.14E-04	7	14385664	14426301	rs16902692	1.08E-03	FRMD4A
10	4	53701449	53715401	rs1194516	3.30E-02	4	53696742	53702708	rs16929092	3.32E-03	PRKG1
10	6	61607010	61624482	rs12355908	3.95E-03	6	61585961	61607010	rs10119177	8.68E-03	ANK3
10	12	97281219	97339124	rs1536444	9.74E-04	8	97286712	97318201	rs7873766	8.41E-03	SORBS1
10	11	127458448	127494379	rs11244664	4.14E-03	4	127471364	127485187	rs7470086	3.52E-03	UROS
10	4	127630157	127646057	rs4403725	1.92E-03	4	127635773	127646057	rs1529192	1.13E-03	FANK1
11	4	20971341	20972871	rs10766761	1.69E-03	7	20959597	20974482	rs1891983	4.11E-03	NELL1
11	6	21530697	21556573	rs4922847	4.62E-03	6	21538074	21567545	rs17158139	1.23E-02	NELL1
11	6	122127741	122146466	rs12804711	8.64E-03	6	122109340	122129879	rs12261326	6.31E-03	UBASH3B
12	4	1854005	1867513	rs4765855	2.27E-02	4	1851711	1860652	rs16920334	1.58E-03	CACNA2D4
12	4	6210649	6216045	rs3181301	3.26E-02	5	6196175	6218155	rs16924415	4.62E-03	CD9
12	6	25136871	25157470	rs7303669	1.02E-02	4	25130689	25143445	rs17703918	2.11E-03	CASC1, LRMP
12	4	93905833	93915516	rs11107845	4.83E-03	4	93899571	93908819	rs3816785	0.016554	NDUFA12
12	6	110129183	110133727	rs3809291	2.21E-02	6	110118664	110143457	rs10829448	3.37E-03	CUX2
13	6	99172061	99202794	rs1125436	2.55E-03	4	99179251	99187733	rs10749902	0.012953	CLYBL
14	9	22081305	22111496	rs1263663	1.19E-02	8	22099352	22112693	rs1078402	5.22E-04	TRA@, TRAC, DAD1
14	12	32287714	32314819	rs910318	3.38E-03	4	32295737	32307125	rs608871	1.48E-03	AKAP6
14	6	56122035	56145673	rs7141305	2.45E-02	4	56129133	56137765	rs216852	1.17E-03	C14orf101
14	9	72786268	72812034	rs7202	7.26E-03	7	72786527	72791053	rs12320955	4.52E-03	PAPLN
14	27	93977822	94053547	rs11626091	2.74E-05	5	94033360	94053376	rs11051219	3.19E-04	SERPINA12
14	4	102682945	102688800	rs719252	1.09E-02	5	102686871	102702856	rs1908592	1.14E-03	RPL21P12
15	4	31741944	31745419	rs4779628	6.64E-03	4	31741944	31750247	rs2232562	2.88E-02	RYR3
15	9	77528074	77560994	rs7169963	6.33E-04	4	77538688	77556997	rs3811170	1.68E-03	KIAA1024
15	7	78050272	78071496	rs1879894	3.85E-03	4	78048881	78059873	rs17685991	3.86E-03	BCL2A1
15	15	87507774	87562668	rs8028123	1.34E-03	4	87510142	87521605	rs1091646	7.37E-04	ABHD2
15	15	87507774	87562668	rs8028123	1.34E-03	4	87562492	87564607	rs12579003	1.91E-03	RLBP1
15	5	91550160	91553882	rs1872052	9.80E-04	6	91532308	91561784	rs11107909	4.23E-03	UNQ9370
15	4	98675178	98686110	rs8029650	1.91E-03	6	98677092	98691878	rs10778338	6.03E-03	ADAMTS17
15	6	99372427	99380316	rs2412004	3.22E-03	4	99375750	99389091	rs4964353	6.79E-03	LRRK1
16	14	79197141	79228642	rs12448290	6.69E-04	4	79205051	79220547	rs7318115	1.17E-03	CDYL2
16	4	81784515	81789713	rs17675933	2.75E-03	17	81783353	81829795	rs9564436	2.34E-04	CDH13
16	6	82535061	82542626	rs2245222	6.97E-03	4	82523937	82537492	rs9599646	4.77E-03	OSGIN1
16	4	83008592	83017058	rs247805	2.20E-02	4	82992730	83011363	rs7329434	2.20E-02	ATP2C2
17	12	28797681	28835589	rs952540	1.74E-04	4	28797681	28806815	rs17502818	1.40E-03	ACCN1
17	12	28797681	28835589	rs952540	1.74E-04	6	28827828	28847221	rs16959573	1.66E-03	ACCN1
17	4	50752149	50762786	rs12453544	8.62E-03	5	50760650	50785227	rs354445	1.95E-03	HLF
18	4	53871218	53884508	rs4941304	4.08E-03	4	53871218	53879450	rs1531634	3.69E-03	NEDD4L
18	4	55281959	55298778	rs12961264	3.31E-03	5	55287091	55289937	rs2293839	7.33E-04	CCBE1
18	4	55317233	55327896	rs7243244	1.90E-02	5	55301972	55322776	rs17111687	9.60E-05	CCBE1
19	4	56810110	56822600	rs4802831	6.81E-03	5	56800494	56823545	rs1402861	9.21E-03	SIGLEC5
20	7	15738136	15756549	rs6135562	2.77E-03	5	15756444	15767911	rs11636091	1.04E-03	MACROD2
20	9	15846030	15864192	rs6034328	4.87E-04	17	15823691	15880560	rs7496492	6.47E-03	MACROD2
20	4	36422510	36431069	rs12624843	3.51E-03	4	36411050	36428083	rs893909	2.23E-02	LBP
20	10	42059032	42099777	rs6031301	1.55E-03	4	42086347	42093700	rs4778721	8.16E-04	TOX2
20	6	51147482	51159764	rs16997525	5.30E-04	6	51153258	51173380	rs1865814	7.16E-05	TSHZ2
21	4	39116086	39121078	rs11254	1.72E-03	23	39116086	39156867	rs9939213	9.26E-04	ETS2
21	5	40051798	40064802	rs8128850	6.09E-03	12	40057196	40094216	rs1528601	5.25E-03	IGSF5
21	10	40503592	40529634	rs447940	8.54E-03	25	40485110	40547818	rs12448529	6.06E-03	DSCAM
21	6	40567354	40589103	rs2837545	2.06E-02	6	40572993	40603541	rs12324955	1.03E-03	DSCAM
21	4	46807080	46811595	rs2839327	4.78E-03	5	46788563	46811098	rs1862751	6.07E-04	DIP2A
22	15	21832478	21835952	rs5759621	2.41E-03	6	21824833	21834959	rs2045925	4.17E-04	RAB36
22	4	43494243	43510219	rs5765930	1.85E-02	5	43494161	43512004	rs13333580	2.50E-02	PRR5

Columns list: chromosome, number of nominally-positive SNPs in dbGAP samples, beginning and end of the chromosomal region identified by clustered nominally-significant associations in dbGAP samples, SNP with the minimal p value found in the region, the p value for the SNP with the minimal p value in this region, similar data for the NIDA/MNB samples, and the gene(s) identified by these clustered SNPs.

### African American samples

45,325 SNPs displayed nominally-significant case *vs* control differences for dbGAP samples from African American individuals. For the NIDA/MNB African-American samples, 83,330 SNPs displayed “nominally significant” t values with p<0.05 from this racial/ethnic group. Permutation testing for the dbGAP African-Americans revealed p = 0.69 for the number of SNPs with nominal case *vs* control p values<0.05 (500 trials).

### Searches for genome wide significance in each African-American sample

We identified case *vs* control p values for χ^2^ results from dbGAP samples and for t test results from NIDA/MNB pooled samples [21]. None of these p values approached the 10^−8^ level deemed necessary for genome wide significance.

### Searches for clustering of SNPs with nominally-significant case vs control differences in each African-American sample

We identified clusters of SNPs that displayed nominally significant, p<0.05 case *vs* control differences for p values from χ^2^ results from dbGAP samples and t test results from NIDA/MNB samples (2026 and 3383 clusters, respectively).

### Searches for chromosomal regions identified by clustered SNPs with nominally-significant case vs control differences in both African-American samples

One hundred twenty nine chromosomal regions were identified by clustered nominally-positive results from both of the two African-American samples. None of 10,000 Monte Carlo simulation trials that each began with random sets of SNPs selected from each of the datasets identified as many overlapping regions as found in the true dataset; hence Monte Carlo p<0.0001. Thus, the null hypothesis that the chromosomal regions identified by both African American samples are found based only on stochastic grounds is nullified by these Monte Carlo data.

However, 199 of 200 permutation trials did provide data that identifies as many chromosomal regions from permutated data as those identified by the real datasets. Thus, the null hypothesis that the chromosomal regions identified by both samples are identified based only on stochastic grounds was not nullified by permutation testing, in ways that suggest that structure in the data may have contributed to the known propensity for permutation testing to overestimate false discovery rates in the presence of such structure [28], [29].

The genes that: a) lie in chromosomal regions identified by data from both African-American samples and b) display the most nominally-significant SNPs are listed in Table 2; the complete list of chromosomal regions identified in this way is listed in Table S2. The fraction of the genome occupied by these results is about 220% of that expected by chance, based on the fraction of the genome occupied by clustered nominally positive results from each of the African American samples *(data not shown)*.

**Table 2:** Chromosomal regions and genes identified by clusters of SNPs that provide nominally-significant differences between individuals dependent on alcohol (dbGAP alcohol dependent *v* ctl) or at least one illegal substance (NIDA/MNB drug dependent v ctl) in subjects of African-American heritage.

	dbGAP alcohol dependent v ctl					NIDA/MNB drug dependent v ctl					
*ch*	*# SNPs*	*bp:begin*	*bp:end*	p_min_ SNP	p_min_	*# SNPs*	*bp:begin*	*bp:end*	p_min_ SNP	p_min_	gene(s)
1	9	28084237	28106641	rs6679432	1.94E-02	4	28085800	28094024	rs17257252	4.41E-03	C1orf38, RPA2
1	5	160357908	160384670	rs1337072	1.36E-02	4	160382664	160395446	rs12124105	1.52E-02	NOS1AP
1	5	182071303	182087392	rs10494570	1.11E-02	7	182079430	182107726	rs11806497	5.41E-04	RGL1
1	4	212649607	212665577	rs10779614	1.19E-02	4	212650752	212655224	rs17022866	2.21E-03	PTPN14
2	4	166129660	166148793	rs10803799	1.16E-04	4	166123345	166130256	rs16850914	3.41E-03	FAM130A2
2	4	240548770	240568964	rs11893710	6.42E-03	4	240542948	240556386	rs13424612	1.19E-03	NDUFA10
3	8	14469859	14483315	rs11128699	6.25E-03	5	14470459	14486703	rs17237132	1.77E-03	SLC6A6
3	4	16380744	16383669	rs9835911	5.03E-03	4	16377563	16389202	rs689953	1.43E-02	RFTN1
3	7	21726437	21747061	rs957589	1.32E-03	8	21717029	21737614	rs13077624	1.02E-02	ZNF385D
3	7	41451566	41467634	rs1495692	4.32E-05	7	41447475	41465298	rs12054014	2.48E-03	ULK4
3	5	62635606	62643369	rs978879	8.10E-03	6	62615383	62637642	rs1512015	1.31E-03	CADPS
3	4	144385643	144403567	rs6776634	3.98E-02	11	144384755	144408513	rs6786129	6.79E-04	PBX2P1
4	9	93699740	93730844	rs7682842	5.98E-04	9	93684320	93699740	rs17319672	8.72E-06	GRID2
4	9	93699740	93730844	rs7682842	5.98E-04	4	93719692	93727390	rs17019608	3.45E-03	GRID2
5	5	7574083	7602448	rs10035541	1.85E-02	7	7591237	7615180	rs1392481	2.56E-03	ADCY2
5	5	41608479	41624460	rs669684	8.81E-03	10	41575032	41617270	rs620876	4.99E-03	TCP1L2
5	4	148176930	148194422	rs12652757	5.08E-03	4	148169604	148179324	rs2116714	2.06E-03	ADRB2
5	4	167355762	167368572	rs17069578	1.99E-03	5	167361210	167387579	rs17069636	2.93E-04	ODZ2
6	4	12225330	12232841	rs2228213	1.71E-02	4	12225541	12239453	rs2327514	2.36E-03	HIVEP1
6	4	31234388	31247483	rs1108746	9.83E-04	4	31230976	31243685	rs9501063	2.86E-03	CCHCR1, POU5F1, TCF19
6	10	32278411	32307330	rs2071287	1.22E-02	6	32288098	32302370	rs2269418	4.43E-04	NOTCH4
6	7	46016169	46032945	rs4714892	5.78E-04	10	45987042	46031767	rs9367228	9.12E-03	CLIC5
6	6	129830351	129839777	rs6569603	1.91E-04	6	129838313	129856864	rs17057464	2.75E-03	LAMA2
6	5	147933198	147957929	rs7743538	6.08E-03	5	147940910	147969453	rs9497816	3.43E-03	SAMD5
6	6	148834310	148848906	rs1124163	3.87E-03	5	148822606	148840150	rs6927662	3.53E-03	SASH1
6	7	168744759	168766408	rs12197584	4.58E-03	4	168765804	168776041	rs9456259	6.38E-03	SMOC2
7	4	37749625	37757420	rs2709114	5.41E-03	6	37754332	37786876	rs2709114	3.52E-03	GPR141
7	10	154494400	154529011	rs1619015	2.17E-03	4	154510872	154528905	rs1730186	1.82E-02	HTR5A
8	5	1466441	1477325	rs17748677	3.40E-03	4	1468372	1478011	rs17681530	3.86E-03	DLGAP2
8	6	3543065	3557725	rs17326670	2.39E-03	6	3537344	3551589	rs17067079	9.79E-06	CSMD1
8	4	4194159	4209292	rs10104910	9.77E-03	4	4200701	4215192	rs3990909	5.21E-03	CSMD1
8	6	17266781	17290933	rs12676388	8.09E-03	4	17281156	17285461	rs7460082	2.31E-02	MTMR7
8	5	72347452	72360941	rs11991562	1.34E-02	8	72351592	72382875	rs6989867	8.03E-04	EYA1
8	4	102824050	102836428	rs6468792	2.12E-02	7	102818744	102835352	rs1125334	2.81E-03	NCALD
8	6	139357180	139371129	rs1512406	5.04E-04	7	139358561	139377143	rs1512407	7.92E-05	FAM135B
8	5	141035897	141054146	rs6981165	3.17E-03	5	141032458	141044168	rs881378	2.54E-03	NIBP
9	4	7155427	7162630	rs10976082	1.43E-02	4	7140997	7157510	rs913581	7.73E-04	JMJD2C
9	4	9408277	9423131	rs4342663	8.69E-03	13	9383744	9435270	rs10816124	7.94E-04	PTPRD, RN7SLP2
9	7	118770530	118810301	rs7042036	3.43E-04	6	118810026	118836028	rs2050274	3.15E-03	ASTN2
10	4	66260831	66274062	rs1227244	1.90E-02	7	66245561	66275840	rs10761866	1.42E-03	ANXA2P3
10	16	74499314	74567822	rs12573512	5.00E-03	9	74540513	74570789	rs6480671	1.23E-03	ECD, NUDT13
10	4	90564031	90575327	rs11817978	1.33E-02	4	90570124	90575118	rs4934423	2.08E-02	ANKRD22, LIPM
10	5	115300542	115311814	rs4918842	1.77E-02	6	115301876	115327383	rs7093962	6.99E-03	HABP2
10	6	135216408	135227438	rs8181425	1.50E-02	4	135209148	135223425	rs9629977	1.94E-03	FLJ44653, SYCE1
11	5	12253357	12271091	rs7106205	1.43E-03	5	12241317	12255138	rs11022270	9.33E-03	MICALCL
11	4	20620979	20632993	rs1617769	2.17E-03	7	20586739	20621980	rs2298826	1.57E-04	SLC6A5
11	4	87727005	87740895	rs4753359	1.68E-03	4	87719881	87730124	rs618143	9.42E-03	CTSC
11	6	88091682	88116810	rs1993842	9.39E-03	5	88095026	88109123	rs2892293	4.54E-03	GRM5
11	4	92259078	92271738	rs9666789	1.52E-02	5	92259808	92275974	rs12421052	2.14E-03	FAT3
11	4	122111119	122121536	rs4935804	2.30E-03	4	122121115	122128473	rs1540113	1.29E-03	UBASH3B
12	5	65221347	65236556	rs10748053	8.18E-04	4	65208873	65229384	rs7971370	4.95E-03	GRIP1
12	4	71010301	71018781	rs10506653	1.09E-02	10	70989581	71025118	rs17783131	6.08E-04	TRHDE
13	17	94683173	94714801	rs4258481	7.94E-03	13	94684855	94734859	rs9590213	1.54E-04	ABCC4
13	5	102501056	102512255	rs157382	1.01E-02	12	102491230	102519109	rs1549836	2.17E-03	SLC10A2
13	4	108262545	108277505	rs9521065	1.24E-03	4	108262052	108276593	rs390790	7.41E-03	MYO16
14	6	85039659	85062024	rs1884009	1.20E-03	4	85043508	85064044	rs17709714	1.19E-03	FLRT2
14	9	85074724	85098018	rs1955418	2.29E-03	4	85086089	85096268	rs985620	1.98E-04	FLRT2
15	4	24767181	24769395	rs28551016	8.86E-04	4	24761221	24768832	rs4887529	1.07E-03	GABRA5
16	4	81469875	81483050	rs16958826	1.42E-03	14	81469724	81505974	rs9319578	5.41E-05	CDH13
19	5	11815562	11851258	rs286246	2.12E-03	4	11816632	11836425	rs1466308	8.54E-03	VN2R13P, VN2R14P, ZNF439, ZNF440
20	4	6702246	6720263	rs235704	5.80E-03	4	6714019	6722420	rs13044579	5.60E-03	BMP2
20	6	19983767	20012701	rs6046593	8.04E-03	5	19997088	20023996	rs9808594	5.69E-04	C20orf26
20	10	48907768	48942090	rs1062651	9.85E-04	4	48912167	48930175	rs6096138	5.45E-03	BCAS4
20	5	54252037	54262859	rs6099057	1.10E-03	7	54233123	54261400	rs6123568	4.10E-03	MC3R
21	7	26422204	26447377	rs12482753	4.85E-03	12	26419368	26465912	rs9984764	1.61E-03	APP
21	6	40435728	40454200	rs2837468	1.36E-02	6	40446501	40459247	rs11911749	3.25E-04	DSCAM
22	6	15695102	15706432	rs165611	1.02E-04	5	15689881	15706432	rs2075120	1.62E-04	CECR8
22	4	29185674	29194610	rs5753158	6.46E-03	10	29169346	29215980	rs5753152	2.18E-04	SEC14L3
22	11	35727496	35747143	rs6000529	1.11E-02	5	35733248	35738081	rs130598	7.85E-03	C22orf33, MPST, TST

Columns list: chromosome, number of nominally-positive SNPs in dbGAP samples, beginning and end of the chromosomal region identified by clustered nominally-significant associations in dbGAP samples, SNP with the minimal p value found in the region, the p value for the SNP with the minimal p value in this region, similar data for the NIDA/MNB samples, and the gene(s) identified by these clustered SNPs.

### Searches for genes identified by clustered SNPs with nominally-significant case vs control differences in all four samples

The clusters from both of the two African-American samples identified six genes that were also identified by clusters from both of the two European-American samples. CDH13, CSMD1 and DSCAM are three cell adhesion molecules that we have identified in many prior studies of addiction vulnerability and/or abilities to quit smoking *(see below)*, while CADPS, MTMR7 and UBASH3B have been identified in fewer prior studies. This modest overlap contrasts with the larger overall overlap between the Affymetrix datasets for the African-American *vs* European American NIDA/MNB samples [21] and the Illumina datasets for the African-American *vs* European American dbGAP samples. In the latter case, we can identify 146 chromosomal regions, 88 of which contain 126 genes, in which overlapping results between the two racial/ethnic groups are found in ways not found by chance in 10,000 Monte Carlo simulation trials (*data not shown*).

### Validation of pooling vs individual genotyping for SNPs whose results provided the clusters

We compared individual *vs* pooled allele frequency estimations for the *ca.* 500 SNPs that displayed minor allele frequencies >0.1 and provided clustered, nominally positive results in data from the NIDA pooled samples. The results from these SNPs displayed mean 0.66 Pearson correlation coefficients between data from pooled and individual genotyping. These correlations were more modest than those identified in validating studies for pooling that used larger ranges of expected allele frequencies. Thus, there was an average 0.19 range of expected values for these genotypes *vs* 0.9 range for the SNPs and pools used in initial studies that validated pooling with these Affymetrix 6.0 arrays) [21].

## Discussion

Genome-wide association data of increasing richness is available for many complex disorders. Several of these GWA datasets contain relatively robust results at “oligogenic” loci that can also be identified, in many cases, by linkage-based approaches [30], [31], [32], [33]. Even moderately secure GWA identification of “polygenic” influences on disease, however, is likely to require replicated data from multiple independent samples.

“Template” analyses seek SNPs that provide “genome wide significance” with the same phase of association in data from multiple independent samples. However, there have been no unanimous criteria for declaring replication of *sets* of data in circumstances in which no SNP achieves this level of statistical significance in each of multiple samples.

We have focused on identification of statistical significance for sets of chromosomal regions that are each identified by sets of nominally-significant SNPs from several independent samples. This approach identifies chromosomal regions and genes that are very likely, as a group, to display *bona fide* association with individual differences in vulnerability to develop dependence on an addictive substance. This overall confidence derives from approaches that address distinct sets of null and/or alternative hypotheses to explain the results obtained. *First*, seeking chromosomal regions in each sample that are identified by at least 4 closely-spaced nominally-positive SNPs addresses the null hypothesis that the results obtained are randomly distributed across chromosomes. This initial process also addresses the alternative hypothesis that the nominally-positive SNPs are identified based on technical problems in correctly assigning allele frequency differences to case *vs* control sample comparisons (or in correctly identifying the true variances for these values). Of course, we would expect to see clustering of nominally-positive SNPs in each sample in regions in which there was either a) linkage disequilibrium between the SNPs studied and between these SNPs and functional variants that influenced addiction vulnerability or b) linkage disequilibrium between these SNPs and stochastic differences in haplotype frequencies in individual samples of cases *vs* those in a single sample of controls that are unrelated to the phenotype. The *second* way in which we seek replication identifies, in independent samples, many of the same chromosomal regions based on their content of clustered, nominally positive SNPs. This comparison addresses the null hypothesis that the clustering observed in each sample derives from stochastic case *vs* control differences in haplotype frequencies rather than case *vs* control differences that are truly related to differences in phenotypes. This comparison also provides additional support for our ability to reject the null and alternative hypotheses relating to assay noise. We thus identify more chromosomal regions and genes based on the overlap between the chromosomal regions identified by data from each sample than we would expect if the only reason for clustering of nominally positive SNPs in each sample was stochastic variation in the frequencies with which blocks of restricted haplotype diversity are found in cases *vs* controls that are unrelated to the phenotype. Availability of data from other recently-reported genome wide association studies also provides a *third* way in which we seek replication, based on identification by the current data, of more of the same genes that were identified in other reports from independent samples and different analyses than we would expect by chance. This comparison also provides additional means for us to refute then null hypothesis that the clustering observed in each sample derives from stochastic case *vs* control differences in haplotype frequencies rather than case *vs* control differences that are truly related to differences in phenotypes. In replicated samples that compared 500 k allele frequencies in alcohol dependent to population control samples, Treutlein and colleagues [15] have used a mixed analytic strategy to identify nine genes. Products of two of these genes, ADH1C and PECR, are likely to play direct roles in alcohol metabolism and thus provide weak candidates for overlap with data from the NIDA/MNB samples. Our current results identify three of the remaining seven genes: CDH13, ERAP and CAST. Based on chance, we should have identified fewer than one of these genes (0.07 genes on average). We have also recently begun analyses of a 500,000 SNP dataset supplied by these authors. We have identified chromosomal regions tagged by clusters of at least 3 SNPs which lie within 25 kb of each other that display nominally-significant case *vs* control differences in this sample, criteria that we have previously used for 500 k datasets. These analyses identify 30 of the genomic regions and 18 of the genes identified by both dbGAP and NIDA/MNB European American samples, providing more than 19 times the amount of overlap expected by chance (Uhl GR, Johnson C, Treutlein J, Cichon S, Ridinger M, Wodarz N, Soyka M, Zill P, Maier W, Moessner R, Gaebel W, Dahmen N, Fehr C, Scherbaum N, Steffens M, Ludwig KU, Frank J, Wichmann HE, Schreiber S, Dragano N, Sommer WH, Leonardi-Essmann F, Lourdusamy A, Gebicke-Haerter P, Wienker TF, Sullivan PF, Nöthen MM, Kiefer F, Spanagel R, Mann K, Rietschel M, *unpublished observations*, 2011).

There are a number of important limitations that come from these samples, these analyses, and from the application of this approach to these datasets. The two distinct null hypotheses both require careful thinking about linkage disequilibrium, since it is easy to confuse data and analyses that bear on linkage disequilibrium among markers that display case *vs* control differences in single samples, the chromosomal regions that such markers label, and the chromosomal regions labeled by such sets of markers in multiple independent samples. Especial difficulties in clarity may arise since we anticipate true positive results that combine differences between cases and controls that are based on linkage disequilibrium among markers that display case *vs* control association with disease and between these markers and the functional allelic variants that provide the variation in gene function that influences phenotype. Without dissecting these differences, it is easy to come to the incorrect conclusion that the method described herein is only detecting the linkage disequilibrium structure and not disease association.

There are other limitations. The NIDA/MNB samples, largely of individuals who were not seeking treatment, were recruited at a single site and compare dependent individuals with heavy levels of substance use to controls with modest or no substance use. These features might provide differences from the dbGAP samples which were recruited at a number of sites from largely treatment-seeking individuals or probands. The dbGAP samples compare alcohol dependent individuals to controls whose levels of illegal substance use do not produce dependence, but might be substantial. To parallel the recently-reported analysis of this data by Beirut and colleagues [16], we have included, in the control group, individuals who smoked significant numbers of cigarettes and/or display DSM or FTND dependence on nicotine. Reanalyses of the data from the dbGAP European American sample after excluding the 376 individuals with FTDN scores >4 and/or DSM nicotine dependence yields overlap with data from NIDA samples that is even stronger than that identified in the main analyses presented here, even though more than ¼ of the “controls” are removed from these analyses (Johnson *et al, unpublished observations, 2010*). Due to the small number of individuals with Asian or Hispanic racial/ethnic backgrounds in this sample, we have excluded them from the present analyses. This exclusion also renders our comparisons different from those used in the recent report of data from many of these same dbGAP individuals [16]. While Monte Carlo simulation tests weigh strongly against the null hypothesis that chance alone accounts for the degree to which the same genes are identified by data from each of the two samples from individuals of the same racial/ethnic background, permutation tests only reach high levels of statistical significance in rejecting this null hypothesis in the European American subjects. Principal components analyses suggest that much of the variance in this data is not due to phenotype or racial/ethnic group (Johnson *et al, unpublished observations, 2010*); such structure might account for the permutation results from the African American data [28], [29]. Based on statistical considerations, the present analyses are likely to provide many false negative results. The power of each of these samples to detect polygenic influences is moderate. The requirement for convergent identification of the same chromosomal region by data from both samples of the same racial/ethnic background provides a likelihood of even more false negative results. Case *vs* control allele frequency differences in the NIDA/MNB samples were genotyped using multiple DNA pools and an Affymetrix 6.0 platform, providing t tests that use information about both mean differences and variances. Case *vs* control differences in the dbGAP samples were assessed using Illumina platform genotyping of individual samples, yielding χ^2^ results without explicit assessment of variance. The requirement that at least 4 nominally-significant SNPs lie within 10 kb of each other cannot be fulfilled in a number of chromosomal regions or in a number of genes in which the density of SNPs is too low to meet this stringent requirement (see Supplement of [14] for list of the genes that cannot be assessed with these criteria using the Affymetrix platform). There are only about ¼ million autosomal SNPs that are shared between the *ca.* 900 K and 1 M autosomal SNPs evaluated by the Affymetrix and Illumina platforms, respectively, further exacerbating this problem in many genomic regions.

Despite these limitations, there is highly-significant overall convergence between two comparisons of NIDA/MNB and dbGAP GWA data from substance-dependent individuals *vs* controls: one comparison in European-American subjects and another comparison in African-American subjects. For each of these comparisons, the degree to which clusters of nominally-positive SNPs identify the same chromosomal regions and genes is never found by chance in up to 10,000 Monte Carlo simulation trials.

This evidence for replication, defined in this fashion, also provides striking contrasts to results from attempts to identify replication (and/or generalization) in other ways. For example, results that seek to identify the extent to which the same SNPs display nominally-significant associations with the same phase in each of these replicate samples within each racial/ethnic group identify about as many SNPs with these properties as expected by chance (*data not shown*).

We have previously reported the apparent success of “nontemplate” analyses that are similar to those used herein when applied to data from four independent case *vs* control samples for bipolar disorder [20]. None of these bipolar *vs* control samples, individually, provided results with genome wide significance. These samples combined data from individual and pooled genotyping using different genotyping platforms. Despite these difficulties, the results of nontemplate analyses provided much more frequent identification of the same genomic regions and genes by clustered, nominally positive SNPs from multiple independent samples in bipolar disorder than we would anticipate by chance.

Studies that focus on identifying “template” same-phase association with genome wide levels of significance in multiple independent samples appear most likely to succeed when oligogenic genetic architecture confers large association signals in each independent sample, when the same SNP sets are studied in each, when the disease exhibits little allelic or locus heterogeneity and when there are good matches between the fine patterns of linkage disequilibrium of the samples being studied. Apparent replication “failures” using this approach could thus relate to a number of features that include associations of modest magnitude, sample-to-sample differences in fine patterns of linkage disequilibrium, different amounts of information provided by markers with population-specific differences in allele frequencies, allelic heterogeneity and locus heterogeneity.

Monte Carlo methods allow us to test the probabilities of chance clustering of nominally-positive SNPs and the chance of convergence between clusters identified in one sample with clusters identified in other samples. Our Monte Carlo approaches deploy an empirical method that uses the existing dataset as a source for randomly selected SNPs for each Monte Carlo trial. The results of these simulations provide strong overall confidence that these sets of results are not due to chance. By contrast, these approaches alone provide unequivocal identification of few individual SNPs or genes. This lack of unequivocal identification of individual SNPs is consistent with polygenic/allelic heterogeneity current working models for the genetic architecture of vulnerability to substance abuse [14], [34]. However, identification of associations at some loci, such as the CDH13 locus, in many independent samples *(see below)* makes it very highly unlikely that this locus does not harbor allelic variants that influence interactions between humans and addictive substances.

Previous analyses that have compared the MNB/NIDA European-American to African-American results have identified genomic regions that are labeled by clustered, nominally-positive SNPs from both samples, supporting roles for some allelic variants that are likely to be old in relation to human history [6], [7], [9], [21]. Data from analyses that combine results from individuals with different racial/ethnic backgrounds also provide suggestive results in regions such as the GABA receptor gene cluster on chromosome 4 for evolutionarily-old variants [16], [35]. Identification in both studies of SNP markers whose allelic frequencies distinguish controls from addicts of different ethnicities supports “common disease/common allele” genetic architecture [36] for part of the genetics of addiction vulnerability. However, the substantially greater convergence, noted here, for data from the same racial/ethnic groups also points to possibly-substantial roles for variants that have been accumulated more recently in human populations that have been more separate until relatively recently.

Genes identified by this work include those in several classes. When we compare the list of genes identified by these samples to functional classes as annotated in Gene ontology (GO) using Biobase, we find the greatest (9.2×10^−9^–1.2×10^−6^) statistical significance for overrepresentation of the genes whose products are involved with the following biological processes: signal transmission (57 observed/28 expected by chance), signaling process (57/28), cell communication (39/16), regulation of cellular process (90/57), regulation of localization (23/7), signaling (68/39), negative regulation of biological process (42/19), regulation of biological process (94/63), biological regulation (100/70) and synaptic transmission (16/4).

CDH13 associations with addiction phenotypes have now been identified in both the four samples studied here and in a number of prior reports. We initially identified associations between substance dependence vulnerability and CDH13 variants in smaller subsets of COGA and MNB samples in studies that utilized earlier microarray types [9], [37]. We and others have subsequently identified such associations in several other samples for addiction-related phenotypes that include: a) vulnerability to substance dependence [12], independent replicated alcohol dependence datasets [15], [19], [38], b) individual differences in acute responses to alcohol administration [39] and c) abilities to quit smoking [24], [40], [41]. Allelic variants in CDH13, a glycophosphoinositol-anchored cadherin that is expressed in neurons that lie in interesting brain circuits, are thus very strong candidates to contribute to addiction-related phenotypes.

The findings presented in the current report thus add the strong evidence for involvement of variants in several individual genes, add to the ongoing consideration of methods for comparing GWA datasets and enhance understanding of genetic underpinnings of human addiction. For addictions, as for many complex disorders, such data provides an increasingly rich basis for improved understanding and for personalized prevention and treatment strategies.

## Supporting Information

Table S1 The complete list of chromosomal regions that a) are identified by data from both European-American samples and b) display the most nominally-significant SNPs (genes listed in Table 1).
(XLS)

Table S2 The complete list of chromosomal regions that a) are identified by data from both African-American samples and b) display the most nominally-significant SNPs (genes listed in Table 2).
(XLS)
